# A new species of Baikal endemic sponges (Porifera, Demospongiae, Spongillida, Lubomirskiidae)

**DOI:** 10.3897/zookeys.906.39534

**Published:** 2020-01-22

**Authors:** Natalia A. Bukshuk, Olga O. Maikova

**Affiliations:** 1 Limnological Institute, Siberian Branch of the Russian Academy of Sciences, Ulan-Batorskaya Str. 3, 664 033 Irkutsk, Russia Limnological Institute, Siberian Branch of the Russian Academy of Sciences Irkutsk Russia

**Keywords:** ITS, mitochondrial IGRs, morphological analysis, *
Swartschewskia
*

## Abstract

This paper reports on a new species of the Baikal endemic sponge (fam. Lubomirskiidae) *Swartschewskia khanaevi***sp. nov.** The description of this species is based on morphological and molecular data (ITS and mitochondrial IGRs). Morphologically, *S. khanaevi***sp. nov.** differs from *S. papyracea* by loose tracts arranged in an irregular network as well as the presence on strongyles of compound spines looking like tubercles densely ornamented with simple spines. Moreover, specimens of *S. khanaevi***sp. nov.** show a peculiar structure of the aquiferous system at the body surface that may be an adaptive trait for environmental conditions. Phylogenetic analysis has revealed that *S. khanaevi***sp. nov.** forms a well-supported (0.99) monophyletic clade with *S. papyracea* and is allocated as its sister taxa.

## Introduction

Baikal is the most ancient and deepest lake on the Earth with the huge water volume. The lake is considered to be 25–30 million years old (Mazepova 1995); its maximum depth is 1641 m, and the volume of water body exceeds 23 000 km^3^ (Mats et al. 2001). Due to these facts, the lake is characterised by minor environmental oscillations. The family Lubomirskiidae represents the most spectacular example of endemic radiation in freshwater sponges under the specific conditions of the great lake. According to molecular phylogeny the endemic family Lubomirskiidae was monophyletic and diverged from Spongillidae (Itskovich et al. 2008; Maikova et al. 2012; Maikova et al. 2017). Existence in a stable environment over a long period of time resulted in the disappearance of gemmulation from the life cycle of Lubomirskiidae (Efremova and Goureeva 1989; Manconi and Pronzato 2002).

At present, 14 species are allocated to the family Lubomirskiidae (Efremova 2001, 2004; Manconi and Pronzato 2019). The actual number of species is most likely underestimated. Comprehensive morphological study of the Baikal sponges revealed at least five forms that showed a constant set of morphological characteristics but could not be related to any known species (Khanaev et al. 2018). In this regard, these forms were suggested to be new species. Additionally, some specimens with uncommon morphology have been described (Weinberg 2005). We also observed several sponge specimens having unusual or transitional traits that interfered with precise species identification in our previous study (Khanaev et al. 2018).

The gaps in our knowledge of Lubomirskiidae morphology and taxonomy concern some aspects in the biology of the Baikal sponges. The absence of gemmules, gemmuloscleres, and parenchymal microscleres, which often contribute to taxonomy, complicates species identification (Manconi and Pronzato 2002). Moreover, the majority of the Lubomirskiidae species were described in the late 19^th^ – early 20^th^ century. The descriptions were limited to the classical taxonomy based on diagnostic morphological characters and were often very brief.

The genus *Swartschewskia* Makuschok, 1927 is clearly segregated from other Lubomirskiidae genera (Dybowski 1880, Swartschewsky 1901, Makushok 1927a). Only *Swartschewskia* is characterised by cortex: an ectosomal skeleton, tangential arrangement of primary tracts and stout bent strongyles as megascleres. The genus includes two species: *S. papyracea* (Dybowski, 1880) and *S. irregularis* (Swartschewsky, 1902). *Swartschewskia papyracea* is widely distributed in the depth range of 1–80 m in Baikal. *Swartschewskia papyracea* morphology was reported in a number of works (Swartschewsky 1902, Makushok 1927a, Rezvoy 1936, Manconi and Pronzato 2002, 2015, 2019; Weinberg 2005, Masuda 2009). On the contrary *S. irregularis* is extremely rare species inhabiting sublittoral zone of Baikal (70–150 m). The species was described based on the single specimen that was not preserved (Swartschewsky 1902). During the next 120 years only one sponge specimen with a similar morphology was found but no data on its morphology were published (Efremova 2001).

Molecular approach is also limited for phylogenetic studies of the Baikal sponges due to low variability of markers (COI, ITS, rRNA-genes) usually applied for this purpose throughout the world (Itskovich et al. 2008, Harcet et al. 2010). Recently (Maikova et al. 2015, 2016), the protein coding sequences of mitochondrial DNA were used to study the phylogenetic relationship of Baikal sponges only at the genera taxonomic level. This study showed that the nucleotide substitution rate of intergenic regions (IGRs) of the Baikal sponge mtDNA is significantly higher than coding sequences, which makes them very promising for phylogenetic reconstructions of closely related species (Lavrov 2010, Maikova et al. 2012). However, only concatenated nuclear (ITS-regions) and mitochondrial (IGRs) data allowed us to separate closely related species of the family Lubomirskiidae (Maikova et al. 2017). Therefore, in this study we use ITS and mitochondrial IGRs sequences to investigate the phylogenetic position of a new species within the family Lubomirskiidae.

During the 2016 expeditions, unusual sponges were sampled in Olkhonskiye Vorota Strait. These sponges were identified as a separate species based on their morphological and molecular phylogenetic data. The paper describes a new species of *Swartschewskia* and we present additional data on the morphology of *Swartschewskia papyracea* (Dybowski, 1880) and provide diagnostic keys for the species belonging to the genus *Swartschewskia*.

## Materials and methods

### Study site and sample collection

**Figure 1.**
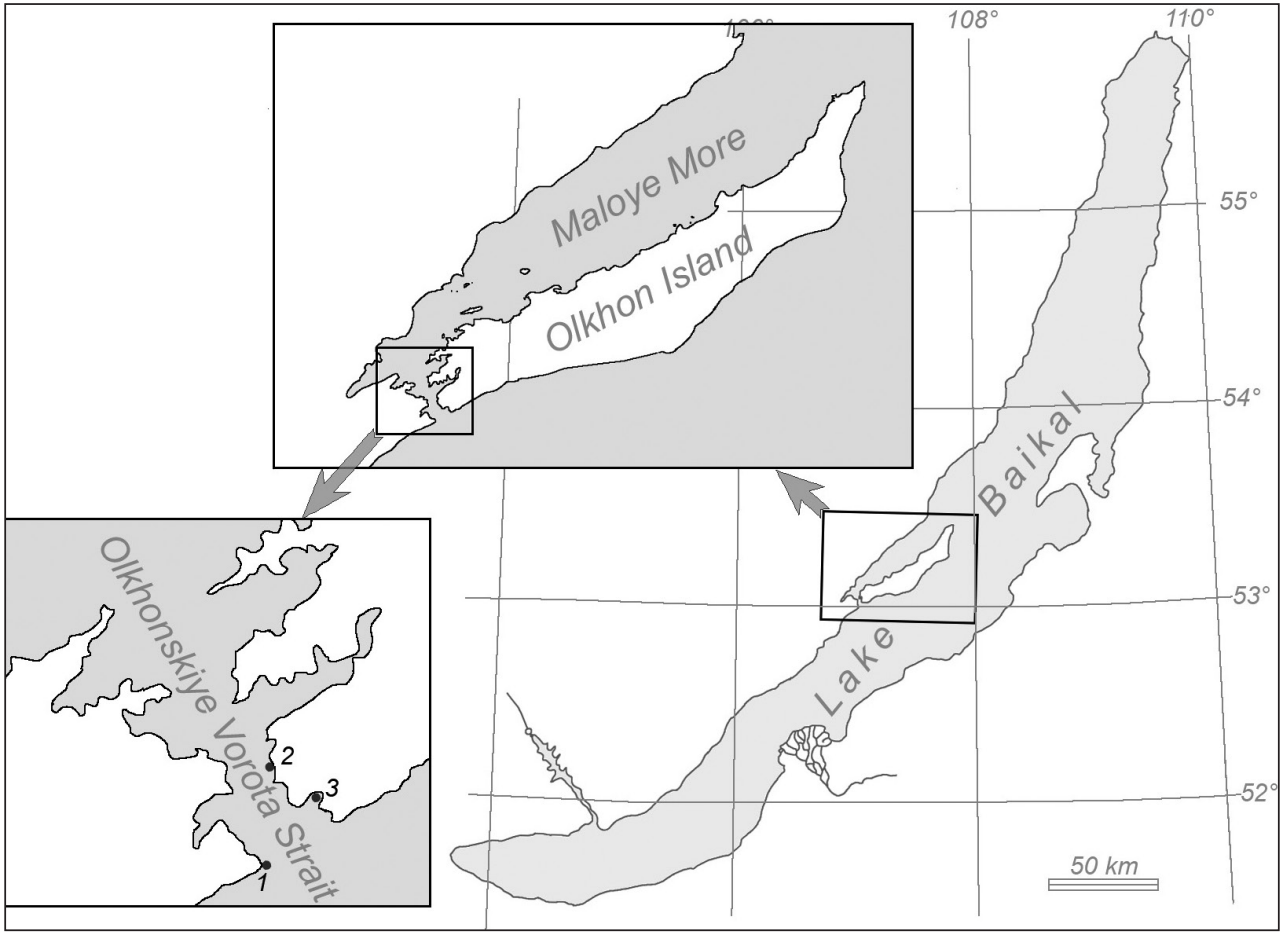
Sampling sites in Lake Baikal: **1**, **2** samples of *S. khanaevi* sp. nov, **3** samples of *S. papyracea*.

The Olkhonskiye Vorota is a narrow strait that connects the Maloye More Strait with the main part of Baikal. The bottom of the Maloye More and the Olkhonskiye Vorota straits consists of different types of ground: rock debris, boulders, pebbles, various sand fractions, and silt (Kozhov 1947). The samples were collected at the three study sites (Fig. 1). At the study site 1 and 2, an extensive multi-layered bank of rock debris is located along the shore from the shoreline to the depth of 4–10 m. Stone fragments have rather large interstices between each other. The interstices are not filled with smaller fractions of ground (such as gravel, sand or silt); hence, unhampered water movements can take place there. Below 10 m, the bottom is sandy with rare boulders submerged in the sand with their lower side. At the study site 3, the bottom mainly consists of sand with detached boulders and rocks, which can be partially submerged in sand.

Samples were collected by SCUBA divers. All specimens were photographed and fixed in 96 % ethanol or frozen at -20 °C.

Holotype and four paratypes of the new species (specimens in ethanol and microscopic slides with tissue-free spicules preparations) have been deposited in Zoological Institute of the Russian Academy of Sciences, St Petersburg (**ZIN**). One paratype has been deposited in Porifera collection of Laboratory of Analytical and Bioorganic Chemistry, Limnological Institute of the Russian Academy of Sciences, Irkutsk (**LIN**).

### DNA extraction, amplification, and sequencing

Total DNA was extracted from approximately 0.1 g of fixed tissue by modified phenol-chloroform method (Maniatis et al. 1984). For molecular analysis, two internal transcribed spacers (ITS1 and ITS2), as well as intergenic regions (IGRs) between tRNA^Tyr^ and tRNA^Met^ genes of mitochondrial DNA (mtDNA), were sequenced. Amplification of ITS and IGRs sequences was performed using specific primers and parameters described previously (Itskovich et al. 2008; Maikova et al. 2012). Each PCR reaction was purified by electrophoresis in 0.8 % agarose gels and eluted by freezing and thawing. DNA sequencing was carried out using the BigDye Terminator V3.1 Cycle Sequencing Kit (Applied Biosystems, United States) with subsequent analysis of the reaction products on an Applied Biosystems 3130xl Genetic Analyzer sequencer (USA) at Syntol Company (Moscow, Russia).

### Sequence alignments and tree reconstructions

PCR-fragments were assembled and aligned using MAFFT (Katoh and Toh 2008) and BioEdit 5.09 (Hall 1999). Bayesian reconstructions were performed using MrBayes v. 3.2.1. (Ronquist and Huelsenbeck 2003). For concatenated data (ITS and IGRs), the nucleotide substitution model GTR+I+G was used for ITS and “mixed” parameter for IGRs. The Markov chain Monte Carlo search was run twice (default parameter) on four chains for 20 000 000 generations. Trees were sampled every 1000 cycles after the first 10 000 burn-in cycles. Genetic distances in pairwise comparisons between all analysed sequences were calculated according to the Kimura’s two-parameter model using MEGA7 (Kumar et al. 2016).

### Morphological analysis^1^

Two variants of skeleton preparations were made for each specimen. In the first case, the small pieces of specimens were saturated with water and frozen. Vertical sections of frozen pieces (0.3–0.5 mm thickness) were made manually to investigate the ectosomal and choanosomal skeleton (Efremova 2004). In the second case, the small piece of the ectosomal skeleton was detached from the choanosomal one, washed with water and oriented upwards with the superior surface. Both kinds of skeleton preparations were mounted on slides and on stubs. Tissue-free spicules preparations were made by dissolving small pieces of sponge in sodium hypochlorite with a subsequent rinse with water and transfer to 96 % ethanol. Clean spicules and skeleton pieces were mounted on slides and aluminium stubs and air-dried. Samples on slides were placed in Canada balsam for the observation with Light Optical Microscope Olympus CX-21. Samples on aluminium stubs were coated with gold for further investigation with a Scanning Electron Microscope Philips SEM 525 (Collective Instrumental Center "Ultramicroanalysis" at LINSBRAS). Digital images of both kinds of skeleton preparations and spicules were made using an integrated camera by SEM. Skeleton preparations were also photographed using the light optical microscope with an ocular camera ToupCam 5.1. Spicules of six samples (length and width; 50 spicules in every sample) were measured in several fields of view using Olympus CX-21 and ocular micrometre. Spicules dimensions were listed as three values: minimum–(mean)–maximum.

Cortex thickness and dermal pores dimensions were measured by Philips SEM 525 digital images in every investigated specimen (*N* = 9). Dermal pores ranged from rounded (diameter was measured) to ovoid or elliptic (width and length were measured). Dimensions were listed as three values: minimum–(mean)–maximum. Light optical microscope photographs were used for pore fields and oscula measurements. Apertures in the sieve-like osculum of *S. papyracea* were measured by photograph (*N* = 1).

The percentage of sponge surface lacking in both oscula and pores was calculated in SpongeArea (original software is available at https://gitlab.com/bukshuk-sci/spongearea). Macro photographs for the analysis were taken using Canon EOS 450D with LPL Copy Stand CS-40.

For the taxonomy of genus and species level and name validity the World Porifera Database was considered as reference (Van Soest et al. 2019).

## Results

### Phylogenetic analysis

For phylogenetic analysis, the ITS and mitochondrial IGRs sequences were obtained from six specimens of *S. khanaevi* sp. nov. and two specimens of *S. papyracea*, which were deposited into GenBank (Table 1). Additionally, we used previously published sequences of Lubomirskiidae and Spongillidae species (Maikova et al. 2017). *Ephydatia fluviatilis* (Linnaeus, 1759) (fam. Spongillidae) was used as an outgroup (Itskovich et al. 2008). The length of the aligned concatenated sequences was 1266 bp, the ITS and IGRs partitions were 734 bp and 532 bp in the length respectively. The overall variability (K2P) for ITS-regions was 1.9 % and for IGRs – 0.9 %. Within the family Lubomirskiidae the intraspecific genetic distances of the ITS-regions varied from 0 to 0.6 %, while the interspecific ones varied from 0.1 to 4.7 % (average 1.5 %). The intraspecific genetic distances of the IGRs varied from 0 to 0.8 % and the interspecific ones varied from 0 to 4.9 % (average 2.2 %). Based on concatenated data the intraspecific genetic variability was from 0 to 1.6 % and between species ones was from 0.3 to 4.2 % (average 1.7 %). The pairwise genetic distances between the sequences of *S. khanaevi* sp. nov. varied from 0 to 1.5 % (average 0.7 %), and the ones between the sequences of *S. khanaevi* sp. nov. and *S. papyracea* ranged from 1.4 to 2.6 % (average 1.9 %).

**Table 1.** Sample numbers in the collection and sequence numbers in GenBank.

**Species**	**Number in the collection**	**GenBank number**	
		**Sequences of ITS-regions**	**Sequences of mtDNA intergenic regions (IGRs)**
*S. papyracea*	LIN-BS-1837	MH133907	MH257749
	LIN-BS-2360	MH133908	MH257750
*S. khanaevi* sp. nov.	ZIN 11990	MH133901	MH257748
	LIN-BS-1740-2	MH133902	MH257744
	ZIN 11986	MH133903	MH257746
	ZIN 11987	MH133904	MH257743
	ZIN 11988	MH133905	MH257745
	ZIN 11989	MH133906	MH257747

**Figure 2.**
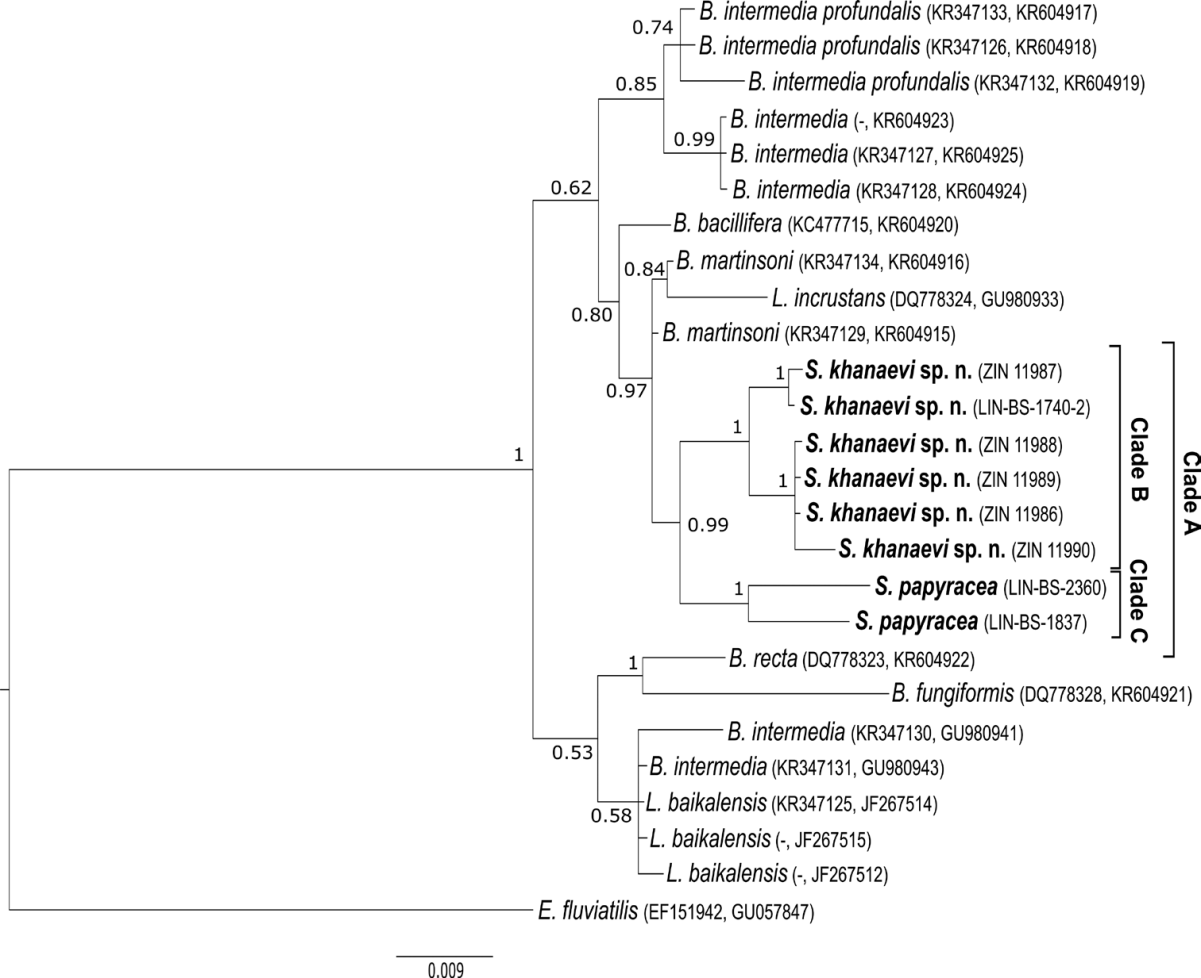
Phylogenetic tree based on concatenated nuclear (ITS1 and ITS2) and mitochondrial (IGRs) sequences: Bayesian posterior probabilities are shown at the bases of the clusters. Taxon names and collection numbers of sponges analysed in this study are marked in bold. Scale bar denotes substitutions per site.

In the phylogenetic tree, the specimens of *S. papyracea* and *S. khanaevi* sp. nov. form a well-supported (0.99) monophyletic clade named A (Fig. 2). Within clade A, we recognise two well-supported monophyletic groups named B and C. Clade B contains the specimens of *S. khanaevi* sp. nov.; *S. papyracea* was allocated as its sister group (clade C). Thus, *S. khanaevi* sp. nov. was named as a separate species.

### Systematics

#### Phylum Porifera Grant, 1836

##### Class Demospongiae Sollas, 1885

###### Subclass Heteroscleromorpha Cárdenas, Pérez & Boury-Esnault, 2012

####### Order Spongillida Manconi & Pronzato, 2002

######## Family Lubomirskiidae (Weltner, 1895)

######### Genus Swartschewskia

Taxon classificationAnimaliaSpongillidaLubomirskiidae

Makushok, 1927

6B82B1DF-DBB6-57D8-A41F-FBD66E04B59D

########## Included species.

*Swartschewskia papyracea* (Dybowski, 1880), *Swartschewskia irregularis* (Swartschewsky, 1902).

########## Type species.

*Swartschewskia papyracea* (Dybowski, 1880).

########## Genus diagnosis.

Body shape encrusting to globose or branched. Ectosomal skeleton hard and well developed as more or less regular alveolar network of thick tangential spicular fibres. Choanosomal skeleton sparsely developed with scarce spicules irregularly arranged in few weak fibres. Abundant spongin. Megascleres strongyles, from spiny to smooth (modified from Manconi and Pronzato 2002).

######### Swartschewskia khanaevi sp. nov.

Taxon classificationAnimaliaSpongillidaLubomirskiidae

1826FDDB-C0FD-55F7-997B-E9B98D34028C

http://zoobank.org/40B1B85A-7E8E-4C09-8DD7-15CE4BB56A28

**Figure 3.**
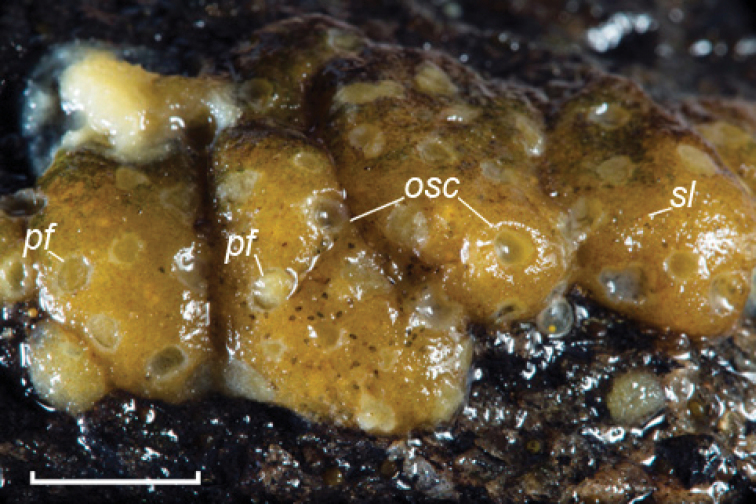
*Swartschewskia khanaevi* sp. nov., external view. Abbreviations: **osc** oscula, **pf** pore fields. Scale bar: 5 mm.

**Figure 4.**
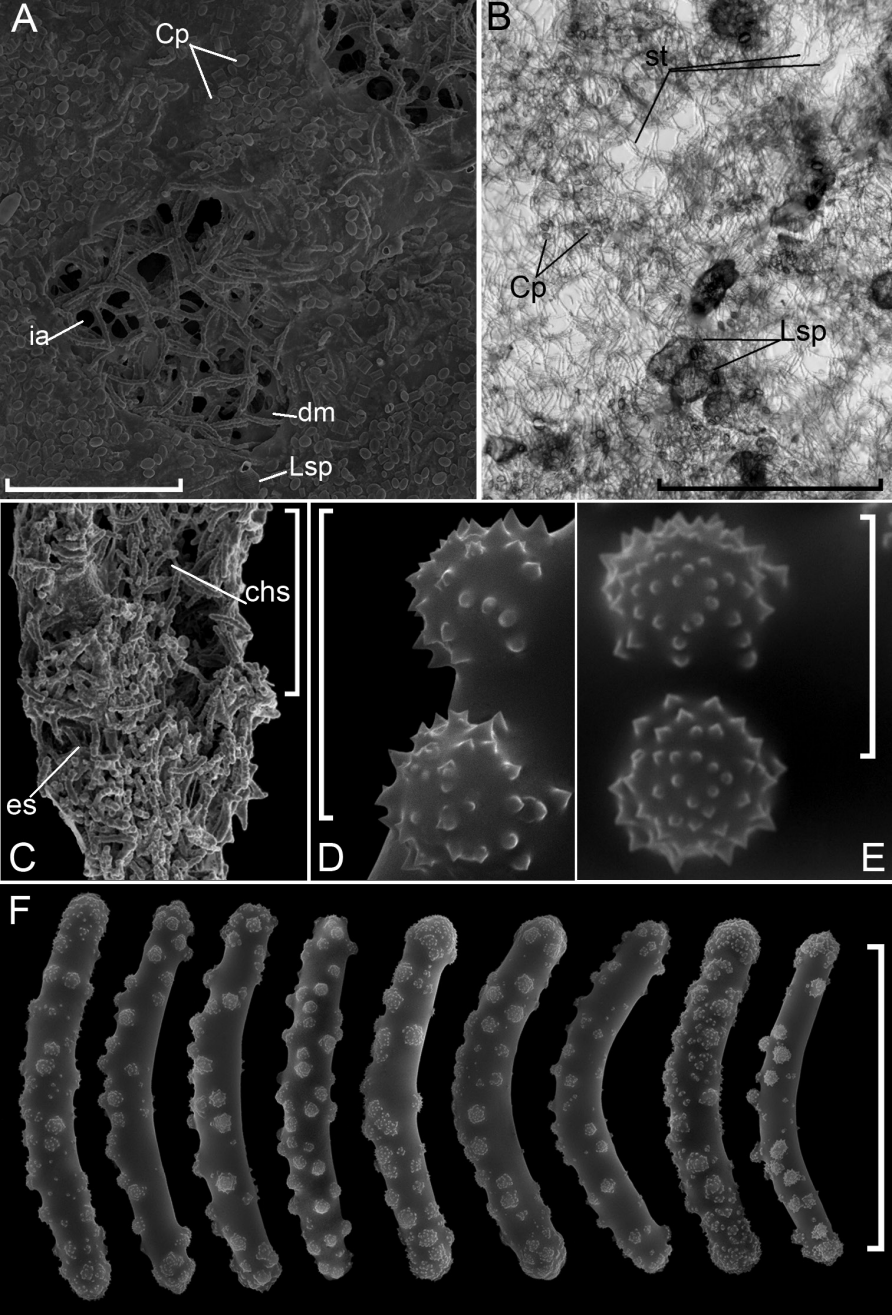
*Swartschewskia khanaevi* sp. nov. **A** sponge surface **B** ectosomal skeleton **C** cross section of skeleton **D, E** secondarily microspined tuberculated spines on strongyles **F** strongyles. Abbreviations: **chs** choanosomal skeleton, **Cp**Cocconeis placentula, **dm** dermal membrane, **es** ectosomal skeleton, **ia** inhalant apertures, **Lsp***Lagenophrys* sp., **st** spicular tracts. Scale bars: 10 μm (**D, E**), 100 μm (**F**), 500 μm (**A**), 1 mm (**B**).

Figs 1 , 3 , 4 ; Tables 1 , 2

########## Type material.

***Holotype***: ZIN 11986 (specimen in ethanol), ZIN 11986A (slide), Lake Baikal, the Olkhonskiye Vorota Strait, sampling site 1 (52°59.42'N 106°55.53'E), depth 10 m, SCUBA divers, September 9, 2016, collected by I. V. Khanaev, 1 specimen. ***Paratypes***: ZIN 11987 (specimen in ethanol), ZIN 11987A (slide): ibid, 1 specimen; ZIN 11988 (specimen in ethanol), ZIN 11988A (slide): ibid, 1 specimen; ZIN 11989 (specimen in ethanol), ZIN 11989A (slide): ibid, 1 specimen; LIN-BS-1740-2 (specimen in ethanol, slide): ibid, 1 specimen. ZIN 11990 (specimen in ethanol), ZIN 11990A (slide): the Olkhonskiye Vorota Strait, sampling site 2 (53°01'03.40"N 106°55'47.00"E), depth 2.5 m, SCUBA divers, June 7, 2016, collected by I. V. Khanaev, 1 specimen.

########## Etymology.

Named after Dr Igor V. Khanaev, scientist and diver who organised a dive program and collected type material.

########## Description.

Thin encrusting sponge. Sponge thickness is maximal in the centre of the body (0.5–1 mm) and minimal at the edge (0.05–0.3 mm).

The natural colour is yellowish beige and almost white in ethanol with brown areas on the surface. Usually, sponges have from one to three oscula, and only paratype ZIN 11990 has six oscula. Oscula are almost round, deepened, edged with well-developed spicular vallum. Oscula size is 146–(585)–978 × 235–(663)–1148 μm. Dermal pores are non-uniformly distributed on sponge surface. They are mostly aggregated in pore fields. Those are not deepened relatively to sponge surface, diverse in shape and can join to each other. Round or ovoid inhalant apertures of 7–(42)–106 × 7–(54)–140 μm in size perforate dermal membrane. The apertures are located in meshes of ectosomal skeleton network. Pore fields size varies significantly: 0.07–(0.5)–1.5 × 0.09–(0.7)–2 mm. One field usually contains 4–40 pores; the maximum number of pores is 78. There are also isolated pores.

Up to 70–80 % of sponge surface is lacking in both oscula and pores and covered with dense accumulations of *Cocconeis placentula* Ehrenberg, 1838 and sporadic exemplars of other diatoms (identified by Dr N.A. Bondarenko). Additionally, some ciliated protozoa of genus *Lagenophrys* von Stein, 1851 (identified by Dr T.Ya. Sitnikova) were observed on all specimen of *Swartschewskia khanaevi* sp. nov.

Sponge surface is a hard but fragile crust, i.e., ectosomal skeleton; the inner part of the body is soft and can be easily detached from the crust. The ectosomal skeleton has a form of a cortical layer (cortex) of tangentially arranged tracts forming an alveolar network. Meshes are disordered; size and shape vary. In some parts of the cortex, meshes are indistinguishable; tracts cross irregularly. Megascleres in tracts are arranged in loose bundles, 2–8 megascleres in every bundle. The thickness of cortex varies significantly from 44 to 307 μm. The thickest cortex is observed near oscula, the thinnest one in the areas of pore fields. The choanosomal skeleton is weak; it consists of separated spicules and thin disordered fibres.

Megascleres are exclusively strongyles of 99–(127)–149 × 9–(15)–21 µm with different sorts of spines: simple spines, rosette spines, and a peculiar sort of spine, secondarily microspined tuberculated spines. The latter look like tubercles (4–9 µm in diameter and 1–5 µm in height) densely ornamented with simple spines (number 13–58) and these are the most abundant sort of spines. Rosette spines are comparatively rare (0.8–(1.4)–3.2 × 1–(1.6)–3.6 μm in size, contain 3–9 simple spines). The length of isolated simple spines and simple spines in both kinds of complex spines is similar: 0.1–(0.4)–0.9 µm. Microscleres absent.

**Table 2.** *Swartschewskia khanaevi* sp. nov. spicules length and width (*N* = 50).

**Specimen**	**Length (µm)**	**Width (µm)**
	**min–(mean)–max**	
**Holotype**		
ZIN 11986	111–(128)–141	11–(15)–20
**Paratypes**		
LIN-BS-1740-2	99–(127)–146	11–(15)–19
ZIN 11987	108–(125)–138	10–(15)–19
ZIN 11988	106–(128)–140	11–(14)–18
ZIN 11989	104–(123)–138	13–(17)–21
ZIN 11990	109–(128)–149	9–(14)–18

######### Swartschewskia papyracea

Taxon classificationAnimaliaSpongillidaLubomirskiidae

(Dybowski, 1880)

2E534DAA-8FF0-546F-9239-84BAA6E8CBB7

**Figure 5.**
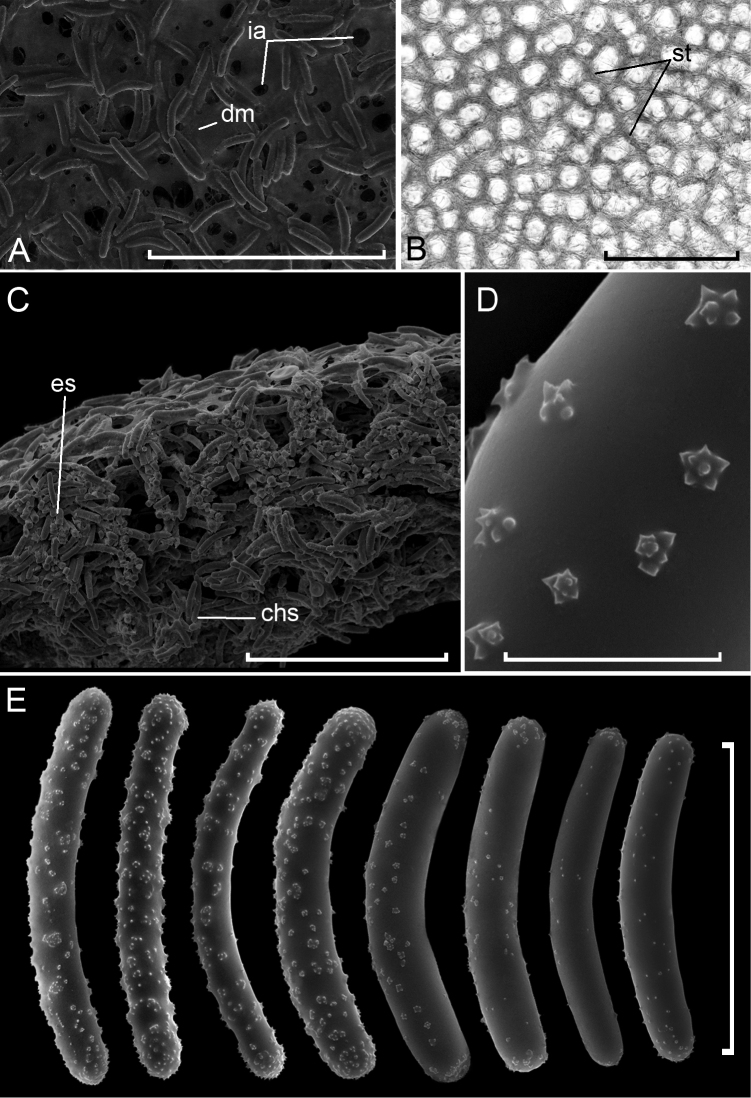
*Swartschewskia papyracea***A** sponge surface **B** ectosomal skeleton **C** cross section of skeleton **D** rosette spines on strongyles **E** strongyles. Abbreviations: **chs** choanosomal skeleton, **dm** dermal membrane, **es** ectosomal skeleton, **ia** inhalant apertures, **st** spicular tracts. Scale bars: 10 μm (**D**), 100 μm (**E**), 500 μm (**A, C**), 1 mm (**B**).

Figs 1 , 5 ; Tables 1 , 2

########## Note.

The morphology of three specimens of *S. papyracea* sampled in the Olkhonskiye Vorota Strait was examined.

########## Description.

Body shape is globose. The sponge often has a single osculum but several oscula are also possible. Mostly the oscula look like round pits with 3–5 exhalant apertures on the bottom. One specimen bears a sieve-like osculum that consists of a number of exhalant apertures not deepened relatively to sponge surface. Distribution of dermal pores is uniform. Inhalant apertures are observed almost in every meshes of ectosomal skeleton network. One mesh contains 1–5 round or ovoid apertures, 5–(28)–87 × 6–(35)–102 µm in size. Exhalant apertures in the sieve-like osculum have elongated or round shape, 214–(281)–357 × 178–(219)–286 µm in size.

The ectosomal skeleton is a high ordered alveolar network, mesh shape resembles a convex polygon. There are no parts with a disordered network structure. Megascleres in tracts are arranged in dense bundles, 6–12 megascleres in every bundle.

Megascleres are stout and bent strongyles of 93–(117)–138 × 13–(17)–22 µm. Analysis of the fine morphological structure of *S. papyracea* spicules indicated the presence of only two sorts of spines: rosette spines and isolated simple spines. Rosette spines are slightly elongated, 0.5–(1.4)–3.2 × 0.6–(1.6)–4.1 µm, and contain 4 – 18 simple spines. Isolated spines and simple spines in rosettes have a similar size of 0.1–(0.4)–0.9 µm.

## Discussion

Two species, *S. papyracea* and *S. irregularis*, were included in the genus *Swartschewskia* before the present study. We used the following sources for comparative analysis of diagnostic morphotraits. The original description of *S. papyracea* was made by W. Dybowski (1880) on several exemplars. Afterwards the type material was lost (Efremova 2001). Due to the impossibility of comparing our data with type material we were guided by the generally accepted recent descriptions of Manconi and Pronzato (2002, 2019). Additionally, we studied the morphology of *S. papyracea* specimens from the type locality of *S. khanaevi* sp. nov. Data on *S. irregularis* morphology are extremely poor. For more than 200 years of studies of the Baikal sponges, only two specimens of *S. irregularis* were collected. Both specimens are no longer available; therefore, we relied on the original description of the species (Swartschewsky 1902).

Based on molecular data, the new species belongs to the genus *Swartschewskia* (fam. Lubomirskiidae). The limitations of the molecular approach were previously shown for phylogenetic studies of the Baikal sponges due to low variability of markers (COI, ITS, rRNA-genes) usually applied for this purpose throughout the world (Itskovich et al. 2008, Harcet et al. 2010). The protein coding sequences of mtDNA allowed phylogenetic relationships within Lubomirskiidae to be resolved only at the genera taxonomic level (Maikova et al. 2015, 2016). The mtDNA intergenic regions, as we suggested, could be suitable for the separation of closely related species of Baikal sponges (Maikova et al. 2012) due to their increased rate of substitution accumulation (Lavrov 2010). But on the phylogenetic tree based on mtDNA intergenic regions, the species of Baikal sponges did not form separate clades at the species level, with some exceptions (deep-sea sponges, for example) (Maikova et al. 2012). The concatenated nuclear (ITS-regions) and mitochondrial (IGRs) data were most suitable for studying phylogenetic relationships within the family Lubomirskiidae at the moment (Maikova et al. 2017). The limitation of the molecular approach is apparently related to the low evolutionary rate of both nuclear and mitochondrial DNA of Baikal sponges (Lavrov 2010), which is enhanced by the relatively recent divergence of many species. An exception is the *S. papyracea*, which shows the acceleration of the accumulation of nucleotide substitutions in mtDNA to be twice relative to other species of the family Lubomirskiidae (Maikova et al. 2015). We hypothesise that this species is one of the most ancient of the existing species. On the phylogenetic tree based on protein-coding mtDNA genes, *S. papyracea* is closer to a common ancestor than all other species of the Baikal sponges (Maikova et al. 2016). In this study, based on concatenated nuclear (ITS) and mitochondrial (IGRs) data, the maximum interspecific genetic distances were between *S. papyracea* and other Lubomirskiidae species. Within the genus *Swartschewskia* the intraspecific and interspecific genetic distances do not overlap. This shows the genetic subdivision of the species within the genus and the genetic isolation of the genus *Swartschewskia* within the family Lubomirskiidae. The division of the new species into two groups inside the Clade B is not reflected in their morphology.

*Swartschewskia khanaevi* sp. nov. has skeleton structure and spicules typical for the genus (Manconi and Pronzato 2002, 2019). *Swartschewskia khanaevi* sp. nov. differs from *S. papyracea* by the clustering of pores in pore fields, less ordered structure of the ectosomal skeleton and unusual secondarily microspined tuberculated spines on strongyles. In *S. papyracea* distribution of dermal pores is uniform, ectosomal skeleton is highly ordered alveolar network with polygonal meshes, spicules bear spines grouped in rosettes (Manconi and Pronzato 2002, 2019). Generally, oscula are similar in both species, but *S. papyracea* has an alternative rather rare kind of osculum, which is sieve-like. It consists of a number of exhalant apertures inside rounded vallum (Manconi and Pronzato 2002). Pore fields of *S. khanaevi* sp. nov. could hardly be misinterpreted as sieve-like oscula. The total shape of the latter is always roundish; apertures are packed very closely and are noticeably larger than dermal pores (mean size 281 × 219 μm vs 42 × 54 μm). Ectosomal skeleton of *S. irregularis* even less ordered than of *S. khanaevi* sp. nov. It lacks polygonal network and looks like randomly arranged spicules. Strongyles of *S. irregularis* are smooth.

Previous data on the morphology of *Swartschewskia* species do not contain records of strongyles ornamented with tuberculated spines or pore fields (Dybowski 1880; Swartschewsky 1901, 1902; Makushok 1927a; Rezvoy 1936; Manconi and Pronzato 2002, 2019). Weinberg (2005) mentioned an unusual specimen of *S. papyracea* as a thin encrusting sponge with numerous oscula and bearing: (a) spicules as stout and bent strongyles thinner than usual in the species and ornamented with massive complex spines; (b) skeleton of clearly divided ectosomal and choanosomal parts, but intensive study of the skeleton was not carried out. The sponge was collected from the Maloye More Strait (the precise locality was unknown). Based on these facts, we suppose that Weinberg met a specimen of *S. khanaevi* sp. nov. Taking into account sites, where *S. khanaevi* sp. nov. was collected, the species is most likely a local endemic of the Olkhonskiye Vorota Strait or Maloye More Strait as a whole.

Fossil spicules similar to *S. khanaevi* sp. nov. were found in the Late Pliocene sediments (interval of 3.2−2.8 Ma) of Lake Baikal (Veynberg 2009). They were described as spicules of extinct species *Palaeoswartschewskia* sp. 1, they were some thinner and longer than in *S. khanaevi* sp. nov. and had complex spines. These spines, with a smaller number of simple spines than tuberculated spines and less expressed tubercles (see Veynberg 2009), represent intermediate variant between rosette spines of *S. papyracea* and tuberculated spines of *S. khanaevi* sp. nov. In this regard, we cannot ascertain the unambiguous identity of *Palaeoswartschewskia* sp. 1 and *S. khanaevi* sp. nov., but these two species are doubtlessly morphologically close.

Non-uniform localisation of pores in *S. khanaevi* sp. nov. is uncommon amongst the Baikal sponges. Normally, in lubomirskiids pores are evenly distributed throughout the sponge surface. The bottom at the study site consists of stones (a substrate for sponges) and sandy areas located nearby. The latter saturate the water with suspended grains of sand. The number of suspended particles combined with hydrodynamic activity can lead to clogging of the aquiferous system (Bell et al. 2015). The concentration of inhalant pores at restricted areas of the body surface was previously described as an adaptive trait of some sponge species living under the conditions of high sedimentation (Rützler 1974; Werding and Sanchez 1991; Pronzato et al. 1998). A larger size of inhalant apertures of *S. khanaevi* sp. nov. in comparison with *S. papyracea* can also prevent clogging.

The presence of sessile ciliates and dense aggregation of diatom algae on the sponge surface is not common for Lubomirskiidae. Isolated diatom algae can be observed sometimes on the lubomirskiids surface. There are no descriptions of mass diatom accumulations on the surface of a number of specimens. Any attached ciliates on sponges in Baikal also have never been mentioned. However, ectosymbiotic sessile ciliates of the Lagenophrys genus were described on Baikal endemic amphipods cuticle (Khalzov et al. 2018). Probably the emergence of a unique epibiotic community on *S. khanaevi* sp. nov. is possible due to unusual structure of aquiferous system. Permanent exhalant and inhalant water currents are normally presented at the sponge surface and prevent its colonisation. In *S. khanaevi* sp. nov., exhalant and inhalant apertures are concentrated in restricted areas. Therefore, up to 80 % of the body surface has no currents, which is a favourable substrate for epibiotic organisms.

### Key to *Swartschewskia* species

The key to Lubomirskiidae genera and species was offered by Manconi and Pronzato (2019) and was the basis for the present key.

**Spongillida: Lubomirskiidae: Genera**

**Table:** 

1	Growth form massive (globular) to encrusting with digitiform outgrowths; consistency firm to hard; surface smooth	**2**
–	Growth form encrusting to massive, branching; consistency soft; densely conulose, variably long conules	*** Rezinkovia ***
2	Megascleres typically strongyles variably spiny	**3**
–	Megascleres typically spiny oxeas	*** Lubomirskia ***
3	Megascleres typically smooth/spiny, stout, bent strongyles **with compound spines**, rare spiny oxeas	*** Swartschewskia ***
–	Megascleres typically smooth strongyles with spiny tips; spiny strongyles and/or smooth/spiny oxeas also present	*** Baikalospongia ***

**Spongillida: Lubomirskiidae: *Swartschewskia* : Species**

Three species are endemic to Lake Baikal.

**Table:** 

1	Massive, rounded or encrusting, bent **spiny** strongyles; rare oxeas regularly spiny	**2**
–	Massive, irregular; bent smooth strongyles	***Swartschewskia irregularis* (Swartschewsky, 1902)**
2	Strongyles with spines in rosettes	***Swartschewskia papyracea* (Dybowski, 1880)**
–	Strongyles with tubercles densely ornamented with simple spines	***Swartschewskia khanaevi* sp. nov.**

## Conclusions

A new species *Swartschewskia khanaevi* sp. nov. was described based on morphological traits and sequences of nuclear (ITS1 and ITS2) and mitochondrial (IGRs) markers. In the molecular phylogeny the specimens of *S. khanaevi* sp. nov. are clustered within a well-defined group containing *S. papyracea* as the most closely related species. Indeed, the specimens’ morphological traits clearly indicate their belonging to *Swartschewskia*: well-developed ectosomal skeleton of tangential spicular fibres and sparsely developed choanosomal skeleton, stout bent strongyles as megascleres. The major morphological traits that distinguish *S. khanaevi* sp. nov. from other congeners are the structure of ectosomal skeleton and compound spines on strongyles. *Swartschewskia khanaevi* sp. nov. was sampled only from the Olkhonskiye Vorota Strait, and we assumed it to be a local endemic of this strait. We suggest the non-uniform localisation of pores on the sponge surface may be an adaptation to biotope conditions.

## Supplementary Material

XML Treatment for Swartschewskia

XML Treatment for Swartschewskia khanaevi

XML Treatment for Swartschewskia papyracea

## Footnotes

In situ photograph of sponges is provided by Viktor Lyagushkin (scale bar is approximate)
